# Metformin Prevents Renal Stone Formation through an Antioxidant Mechanism In Vitro and In Vivo

**DOI:** 10.1155/2016/4156075

**Published:** 2016-10-03

**Authors:** Xiong Yang, Hao Ding, Zhenbang Qin, Changwen Zhang, Shiyong Qi, Hongtuan Zhang, Tong Yang, Zhen He, Kuo Yang, E Du, Chunyu Liu, Yong Xu, Zhihong Zhang

**Affiliations:** Department of Urology, Tianjin Institute of Urology, The Second Hospital of Tianjin Medical University, 23 Pingjiang Road, Hexi District, Tianjin 300211, China

## Abstract

Oxidative stress is a causal factor and key promoter of urolithiasis associated with renal tubular epithelium cell injury. The present study was designed to investigate the preventive effects of metformin on renal tubular cell injury induced by oxalate and stone formation in a hyperoxaluric rat model. MTT assays were carried out to determine the protection of metformin from oxalate-induced cytotoxicity. The intracellular superoxide dismutase (SOD) activities and malondialdehyde (MDA) levels were measured in vitro. Male Sprague-Dawley rats were divided into control group, ethylene glycol (EG) treated group, and EG + metformin treated group. Oxidative stress and crystal formations were evaluated in renal tissues after 8-week treatment. Metformin significantly inhibited the decrease of the viability in MDCK cells and HK-2 cells induced by oxalate. Besides, metformin markedly prevented the increased concentration of MDA and the decreased tendency of SOD in oxalate-induced MDCK cells and HK-2 cells. In vivo, the increased MDA levels and the reduction of SOD activity were detected in the EG treated group compared with controls, while these parameters reversed in the EG + metformin treated group. Kidney crystal formation in the EG + metformin treated group was decreased significantly compared with the EG treated group. Metformin suppressed urinary crystal deposit formation through renal tubular cell protection and antioxidative effects.

## 1. Introduction

International epidemiological and clinical data have recently suggested that the incidence and prevalence of kidney stone is increasing [1–3]. Most renal stones are principally composed of calcium oxalate (CaOx). The formation of human idiopathic CaOx stones in the kidney is the most common pathological condition involving hyperoxaluria [1]. Urinary oxalate excretion is lower in healthy subjects compared with idiopathic CaOx renal stone patients [4]. Furthermore, accumulating studies suggest that oxalate is associated with renal tubular cell injury and stone formation depends on the response of renal tubular epithelium to oxalate with significant free radicals involvement [5–7]. Evidence from clinical and experimental investigations suggested that reactive oxygen species- (ROS-) induced renal tubular cell injury contributed importantly to CaOx stone formation [8].

As such, previous studies have documented that antioxidants such as quercetin, vitamin E, and taurine can protect renal epithelial cells from oxidative injury induced by oxalate and ameliorate crystal deposition in rat hyperoxaluria induced renal stone models [9–11]. However, it is reported that increased levels of antioxidants could not remove oxidative stress easily in clinical trials [12, 13]. It seems that the best approach for decreasing oxidative stress is by targeting the enzyme responsible for the generation of ROS, perhaps targeting nicotinamide adenine dinucleotide phosphate NADPH oxidase which is a major source of ROS in the kidneys by treatment of inhibitors of NADPH oxidase. Thus, we chose metformin, a potent inhibitor of ROS production from NADPH oxidase [14], as an experimental material with which to detect the kidney crystal formation preventing effect. It is well known that metformin is a drug widely prescribed to treat patients with type 2 diabetes and metabolic syndrome. However, the real and potential benefits of metformin therapy go beyond its prescribed usage, including reduced risk of cancer, increased antioxidant protection, and prolonged lifespan [14–16].

In fact, numerous in vivo and in vitro studies showed that metformin is a potent antioxidant that directly suppresses ROS production, scavenges free radicals, and changes antioxidant defense pathways [17–19]. Besides, recent publications have reported that metformin inhibits injuries caused by oxidative stress in renal tubular cells and renal tissues [14, 17, 18]. The initial investigations that motivated our study were to determine whether metformin would prove useful in treating renal injury induced by oxalate or kidney crystal formation.

In the current study, we investigated the effects of oxidative stress induced by oxidase on renal tubular cell and rat kidney tissues, either with or without metformin treatment. Furthermore, we evaluated the amelioration of kidney crystal formation by metformin in an ethylene glycol- (EG-) induced rat stone model.

## 2. Materials and Methods

### 2.1. Cell Culture

MDCK, a canine renal distal tubular epithelium cell line, was obtained from Research of the Chinese Academy of Medical Sciences (Shanghai, China). HK-2, a human renal proximal tubular epithelium cell line, was obtained from Research of the Chinese Academy of Medical Sciences (Beijing, China). Cells were grown in DMEM (Gibco) supplemented with 10% fetal bovine serum (FBS) (Gibco), 4.5 mM glucose, 100 IU/mL penicillin, and 100 *μ*g/mL streptomycin in a 37°C incubator with 5% CO_2_.

### 2.2. MTT Assay

Viability of MDCK cells and HK-2 cells was determined by 3-[4,5-dimethylthiazol-2-yl]-2,5-diphenyltetrazolium bromide (MTT) (Sigma-Aldrich) assay after various treatments. Cells were seeded in 96-well plates until 70% to 80% confluence. Subsequently cells were exposed to various concentrations of sodium oxalate (Sigma-Aldrich) (0, 0.05, 0.1, 0.2, 0.4, 0.8, 1.6, 3.2, and 6.4 mM) and metformin (Sigma-Aldrich) (0, 0.5, 1, 2, 4, 8, 16, 32, and 64 mM) for 2 days. MTT (0.5 mg/mL) in phosphate buffered saline was added and cells were incubated for an additional 4 h at 37°C. At the end of the incubation period, the medium containing MTT was removed, and the formazan crystals so obtained were lysed in dimethyl sulfoxide (DMSO). The optical density was measured using a microplate reader at 490 nm (SpectraMax Plus384, Molecular Devices, USA) and the cell viability was expressed as a percentage of the control culture (100%). To further assess the effect of metformin on MDCK cells and HK-2 cells treated with oxalate, cells were treated with 1.6 mM sodium oxalate for 1 day with various concentrations of metformin and MTT assays were performed to determine cell viability.

### 2.3. Assessment of SOD and MDA Levels In Vitro

After treating MDCK cells and HK-2 cells with 1.6 mM sodium oxalate for various time points (1, 2, and 3 hours) with or without 8 mM or 1 mM metformin, respectively, the intracellular superoxide dismutase (SOD) activities and malondialdehyde (MDA) levels were detected using commercial kits based on colorimetric methods (Nanjing Jiancheng Bioengineering Institute, Nanjing, China). After treatment, MDCK cells and HK-2 cells were harvested by centrifugation and the supernatants were removed. The remaining cells were washed with PBS twice, dissolved in physiological saline solution, and then lysed for 30 min at 4°C. Following centrifugation, the resulting suspension was collected to measure the activities of SOD and the levels of MDA using a different commercial kit according to the manufacturer's instructions.

### 2.4. Animal Model and Experimental Design

Healthy male Sprague-Dawley rats (180–220 g) were used and kept under standard laboratory conditions at temperature (25 ± 2°C), with constant humidity of 55 ± 5% and a 12 h light/12 h dark circle. Animals were randomly divided into three groups, including group 1, normal controls, group 2, EG treated, and group 3, EG + metformin treated, each consisting of six rats. Group 1 was treated with free access to standard rat chow and distilled water for the whole 8-week study period. Groups 2 and 3 rats were fed standard rat chow administered with 0.75% (vol/vol) EG in drinking water for 8 weeks to induce CaOx deposition in kidneys [20–22]. Besides, group 3 rats were treated with metformin dissolved in water by oral gavage at 200 mg/kg/day throughout the 8-week experimental period [23, 24]. At 24 hours before sacrifice the 8-week rats were transferred to metabolic cages and 24-hour urine was collected. Blood and kidney sections were collected at the end of 8-week treatment from 6 rats per group, following the induction of anesthesia by an intraperitoneal injection of pentobarbital sodium (40 mg/kg body weight) (Sigma-Aldrich). For kidney extracting, the right unilateral kidney specimens were stored at −70°C until the following analyses, and the contralateral specimens were fixed with 10% formaldehyde and embedded in paraffin.

### 2.5. Measurement of SOD and MDA Levels In Vivo

SOD activity and MDA content in the kidney tissues samples were determined using commercial kits (Nanjing Jiancheng Bioengineering Institute, Nanjing, China) according to the respective manufacturers' protocols.

### 2.6. Serum and Urinary Biochemistry

The concentrations of calcium in urine and phosphate (P), calcium (Ca), magnesium (Mg), and creatinine levels in serum were determined on a routine autoanalyzer system (Mindray BS-2000M, Shenzhen, China). Urinary pH and volume were measured manually. Urinary oxalate was measured with commercially available kits as the manufacturer's instructions (Trinity Biotech, St. Louis, MO, USA).

### 2.7. Detection of Kidney Crystal Formation

Paraffin-embedded kidney tissue samples were cut into 4 *μ*m sections and stained with hematoxylin and eosin (HE). The sections were analyzed by polarized light optical microphotography to determine renal crystal number and distribution. The number of urinary crystals per high-powered field was counted at 10 random fields (×400 magnification) and graded using a modification of the method as described previously, that is, 0: no crystal, 1: few crystals (1 to 9), 2: a moderate number of crystals (10 to 24), 3: frequent crystals (25 to 49), 4: abundant crystals (50 to 99), 5: very abundant crystals (100 to 199), and 6: closely packed crystals (200 or greater) [7, 10]. Crystallization in each kidney section was quantified by calculating the ratio (percent) of the area containing crystals to low-powered field at 10 randomly selected fields (×100 magnification) using Image Pro Plus (Media Cybernetics, Inc., Bethesda, MD), as described previously [25, 26]. For each kidney, two representative HE stained paraffin sections were prepared on a slide and the average number of crystals deposited was calculated by two independent examiners.

### 2.8. Statistical Analysis

All statistical analyses were conducted using IBM SPSS, version 20. Two-tailed unpaired *t*-test or one-way ANOVA was performed for statistical analysis. Data in graphs and tables are expressed as means ± standard deviation (SD). Differences were considered statistically significant at *P* < 0.05.

## 3. Results

### 3.1. Metformin Protected Renal Tubular Cells from Oxalate-Induced Cytotoxicity

**Figure 1:**
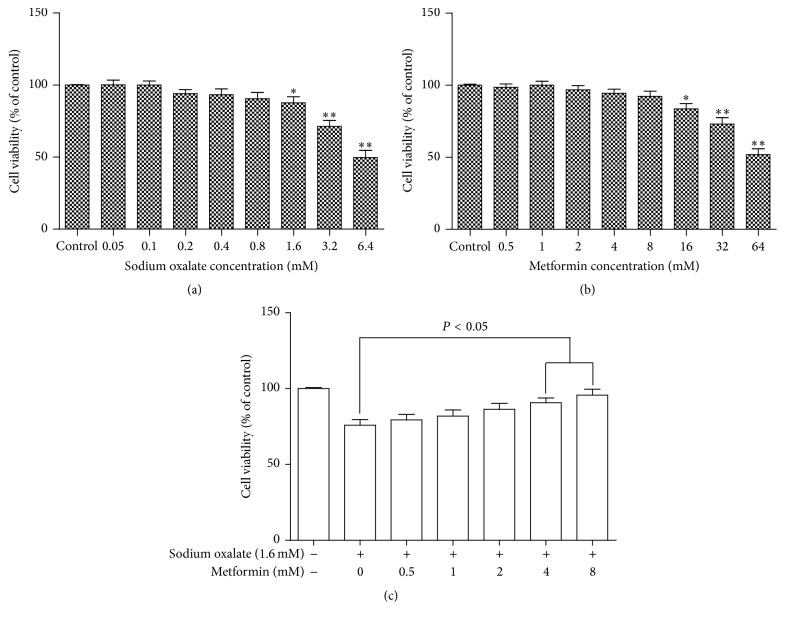
Effect of various concentrations of oxalate and metformin on cytotoxicity in cultured MDCK cells. (a) MDCK cells were treated with the indicated concentrations (0.05–6.4 mM) of sodium oxalate for 2 days. (b) MDCK cells were treated with the indicated concentrations (0.5–64 mM) of metformin for 2 days. (c) MDCK cells were pretreated with different concentrations of metformin (0, 0.5, 1, 2, 4, and 8 mM) for 2 h and then incubated with or without 1.6 mM sodium oxalate for an additional 24 h. Viability of cells was assessed by MTT assay. Percentage of cell viability was relative to the untreated control cells. Values represent mean ± SD of 3 independent experiments. ^*∗*^ *P* < 0.05; ^*∗∗*^ *P* < 0.001, versus control.

**Figure 2:**
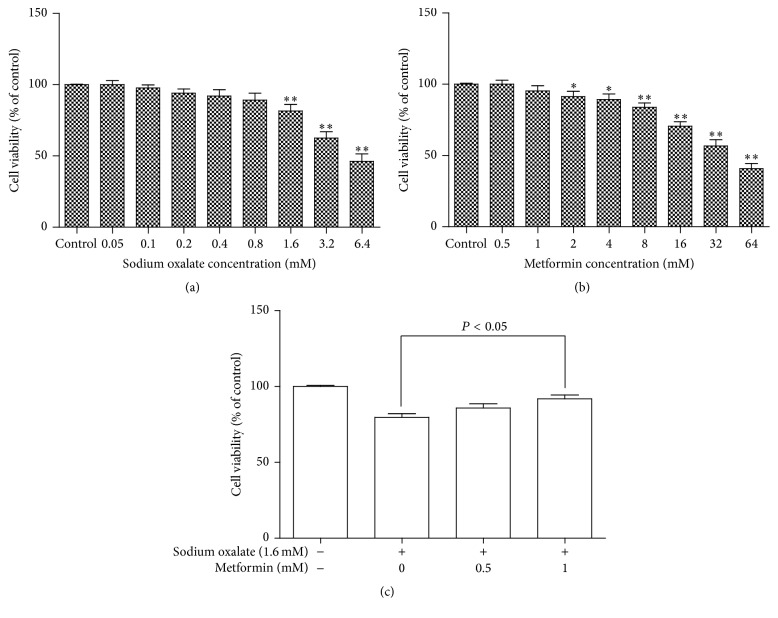
Effect of various concentrations of oxalate and metformin on cytotoxicity in cultured HK-2 cells. (a) HK-2 cells were treated with the indicated concentrations (0.05–6.4 mM) of sodium oxalate for 2 days. (b) HK-2 cells were treated with the indicated concentrations (0.5–64 mM) of metformin for 2 days. (c) HK-2 cells were pretreated with different concentrations of metformin (0, 0.5, and 1 mM) for 2 h and then incubated with or without 1.6 mM sodium oxalate for an additional 24 h. Viability of cells was assessed by MTT assay. Percentage of cell viability was relative to the untreated control cells. Values represent mean ± SD of 3 independent experiments. ^*∗*^ *P* < 0.05; ^*∗∗*^ *P* < 0.001, versus control.

After incubation with various concentrations of sodium oxalate or metformin for 2 days, the cell viability of the cells was determined using the MTT dye reduction assay which is dependent on the activity of mitochondrial enzyme, succinate dehydrogenase in viable cells. As shown in Figures 1(a) and 2(a), cell viability detected by MTT revealed that the number of cells in both cell lines decreased as the oxalate concentration increased. At a concentration of more than 0.8 mM sodium oxalate cell viability in both cell lines decreased significantly relative to control. Metformin had no toxic effect on the MDCK cells and HK-2 cells at a concentration of less than 8 mM or 1 mM, respectively. However, it induced cell viability inhibition at 8 mM or more in MDCK cells and at 1 mM or more in HK-2 cells, as presented in Figures 1(b) and 2(b). More importantly, to investigate the protective effect of metformin on oxalate-induced cell injury, cell viability was tested. Cells were pretreated with various concentrations of metformin (0, 0.5, 1, 2, 4, and 8 mM for MDCK cells; 0, 0.5, and 1 mM for HK-2 cells) for 2 hours before being coexposed to 1.6 mM sodium oxalate for another 24 hours. Cell viability was monitored by the MTT assay, and representative dose-response viability data were illustrated in Figures 1(c) and 2(c). It is noteworthy that this protected effect was significant at a metformin concentration of 4 mM or more in MDCK cells and 1 mM in HK-2 cells.

### 3.2. Metformin Protected Renal Tubular Cells from Oxalate-Induced Oxidative Stress

**Figure 3:**
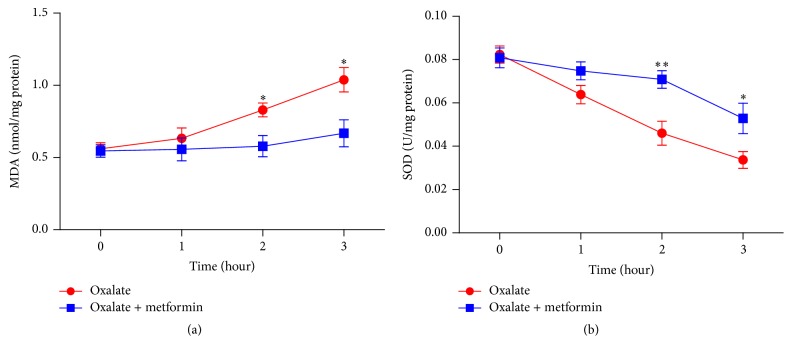
Effect of metformin on SOD and MDA levels in cultured MDCK cells treated with oxalate. (a) MDA levels of MDCK cells were treated with 1.6 mM sodium oxalate with or without 8 mM metformin for the indicated time points. (b) SOD activities of MDCK cells were treated with 1.6 mM sodium oxalate with or without 8 mM metformin for the indicated time points. Values represent mean ± SD of 3 independent experiments. ^*∗*^ *P* < 0.05; ^*∗∗*^ *P* < 0.001, versus control.

**Figure 4:**
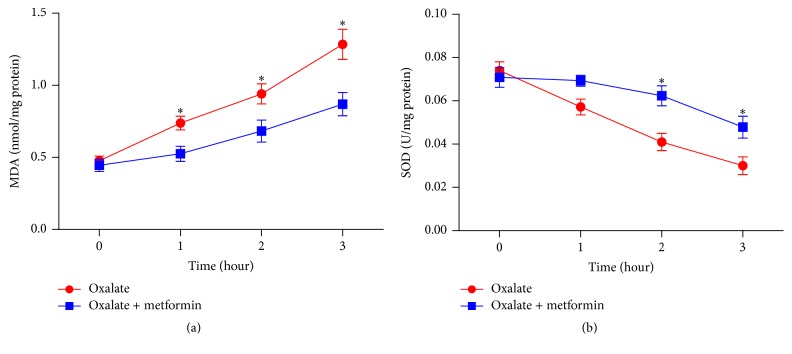
Effect of metformin on SOD and MDA levels in cultured HK-2 cells treated with oxalate. (a) MDA levels of HK-2 cells were treated with 1.6 mM sodium oxalate with or without 1 mM metformin for the indicated time points. (b) SOD activities of HK-2 cells were treated with 1.6 mM sodium oxalate with or without 1 mM metformin for the indicated time points. Values represent mean ± SD of 3 independent experiments. ^*∗*^ *P* < 0.05, versus control.

In order to evaluate whether metformin protects MDCK cells and HK-2 cells form oxidative stress induced by sodium oxalate, SOD activity and MDA content in both 1.6 mM sodium oxalate treated cell lines following different time points (1, 2, and 3 hours) were detected with or without metformin in the present study. MDA levels increased in a time dependent manner after sodium oxalate exposure. When metformin was cotreated, MDA content induced by sodium oxalate was decreased significantly at 2 and 3 hours in MDCK cells and at 1, 2, and 3 hours in HK-2 cells (*P* < 0.05), as shown in Figures 3(a) and 4(a). Respectively, exposure of the cells to oxalate elicited remarkable reduction of SOD activity versus that in untreated controls in a time dependent manner. When the cells were coexposed to oxalate with metformin, the activity of SOD in both cell lines was significantly restored compared to those treated with oxalate at 2 and 3 hours (*P* < 0.05), as presented in Figures 3(b) and 4(b). These experimental findings demonstrated that metformin pretreatment could markedly reduce the oxalate-induced oxidative stress and increase endogenous antioxidant capacity in both MDCK cells and HK-2 cells.

### 3.3. Metformin Relieved EG-Induced Oxidative Stress in Animal Model

**Figure 5:**
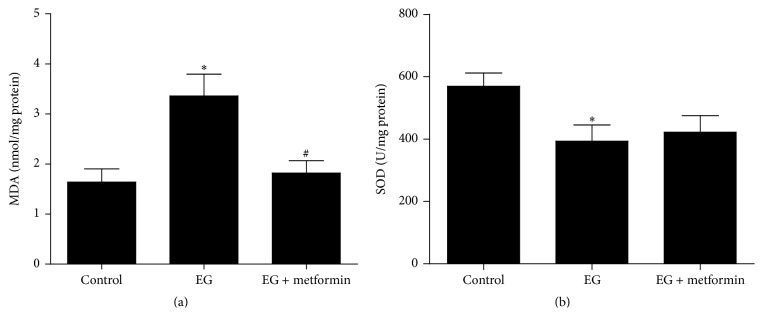
Quantitative assessment shows that MDA level and SOD activity in renal tissue. (a) MDA level and (b) SOD activity. ^*∗*^ *P* < 0.05, versus the control group; ^#^ *P* < 0.05 versus the EG treated group.

To evaluate the lipid peroxidation state of the kidney in the EG-induced rat model, we measured the levels of MDA and SOD in renal tissues. As shown in Figure 5(a), our data showed a significant elevation of MDA levels in the EG treated group compared with controls (*P* < 0.05), while this parameter decreased significantly in the EG + metformin treated group, compared to the stone forming group (*P* < 0.05). In contrast, renal SOD activity was remarkably decreased in the EG treated group versus that in untreated controls (*P* < 0.05), as presented in Figure 5(b). Although SOD activity also slightly decreased in the EG + metformin treated group compared to controls, it was not statistically significant. Besides, there was no significant difference between the EG treated group and the EG + metformin treated group in SOD levels.

### 3.4. Serum and Urinary Biochemistry Results

As shown in Table 1, there were no statistical differences among the 3 groups in serum Ca, Mg, or creatinine. Serum P in the EG treated group was significantly higher than in the control group (*P* < 0.05) but not statistically different between the EG treated group and the EG + metformin treated group. Urinary volume and pH were not significantly changed in any group. The urinary pH level was slightly lower in the EG treated group and the EG + metformin treated group compared with the control group but this was not significant. Urinary Ca in the EG + metformin treated group was significantly lower compared with the control group but there was no statistical difference versus that in the EG treated group. Urinary Ox excretion in the EG treated group and the EG + metformin treated group was significantly higher than in the control group after 8-week administration (*P* < 0.05). Moreover, the EG + metformin treated group showed a significant reduction in urinary oxalate excretion compared with the EG treated group (*P* < 0.05).

### 3.5. Metformin Ameliorated EG-Induced Renal Crystal Formation in Rat Model

**Figure 6:**
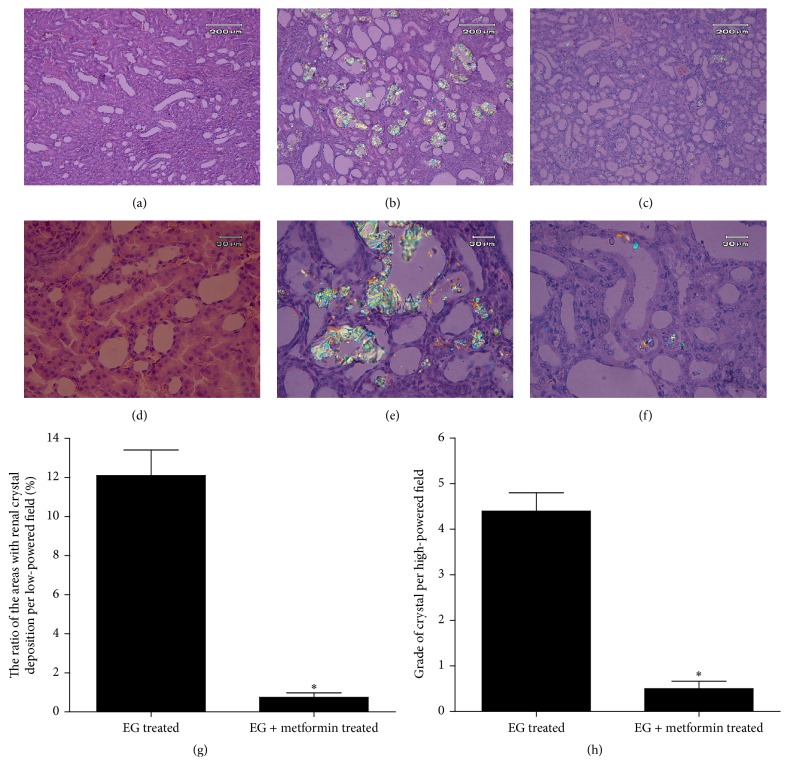
Morphologic distribution and quantitative estimation of renal CaOx crystals. ((a)–(c)) Representative micrographs of renal sections and crystal deposits were presented in the control group, the EG treated group, and the EG + metformin treated group, respectively, using HE staining and polarized light optical microphotography. Original magnification, ×100 in inset. ((d)–(f)) Representative micrographs of the control group, the EG treated group, and the EG + metformin treated group, respectively. Original magnification, ×400 in inset. (g) The ratios of areas with renal crystal deposition per low-powered field were estimated. (h) Grade of calcium oxalate deposits per high-powered field was assessed. ^*∗*^ *P* < 0.05, versus the EG treated group.

No kidney crystals were found at any microscope field in the control group (data not shown). The microscopic analysis of kidney tissue sections under the polarized light showed renal crystal deposits were mainly detected in renal tubules at the border between the renal cortex and medulla, as presented in Figures 6(a)–6(f). Crystal deposition in kidneys was evaluated using a grading system and quantitative methods as described in Section 2. Quantitative analysis of the ratio (percent) of the area containing crystals to low-powered field at 10 randomly selected fields (×100 magnification) showed a dramatic increase in crystal formation in the EG treated group compared to the EG + metformin treated group (*P* < 0.05) (Figure 6(g)). Moreover, metformin treatment markedly decreased the number and grade of renal crystal deposits per 10 fields compared with the stone forming group (*P* < 0.05) (Figure 6(h)).

## 4. Discussion

Recent epidemiological studies have revealed an increased frequency of kidney stone disease all over the world [1–3]. Approximately 80% of the kidney stones contain CaOx as the major mineral composition [27]. To unravel the complexity of the pathogenesis of kidney stone formation, extensive efforts had been undertaken though examinations of clinical data and experiments of animal models and tissue cultures. Current evidence indicates that the probability of CaOx stone formation is directly proportional to increased urinary excretion of oxalate and hyperoxaluria [5]. In fact, previous experiments in vivo and in vitro reported that high concentrations of oxalate were often involved in the onset and progression of renal epithelium injuries as a result of increased intracellular oxidative stress [6]. Similarly, ROS-induced damage of renal epithelial cells and animal models was proposed to have a relevant role in calcium oxalate kidney stone formation [8]. Therefore, various studies reported on the benefits of antioxidants which can decrease the oxidative cellular injury caused by oxalate and inhibit calcium oxalate crystal deposition in the rat kidney [9–11].

Metformin, a potent inhibitor of NADPH oxidase and the most common treatment of type 2 diabetes mellitus, has several properties, such as life-extending capabilities and antitumour activity [15, 16]. Recent investigations strongly showed that metformin prevented oxidative stress-induced injuries in several cell types and tissues, including renal tubular cells and renal tissues [14, 17–19]. However, as far as we know, no study demonstrated the effect of metformin on renal injury induced by oxalate or on kidney crystal sedimentation.

SOD, one of the most important physiological antioxidants against free radicals, could prevent subsequent lipid peroxidation, which serve as a detoxifying system to prevent damage caused by oxidative stress. On the other hand, MDA, an indicator of lipid peroxidation, is one of the primary events in oxidative cellular damage and reflects a status in which ROS is overproduced. Our data showed that metformin considerably prevented the increased concentration of MDA and the decreased tendency of SOD in oxalate-induced renal tubular cells. These findings corroborate those of earlier studies demonstrating that metformin significantly attenuates renal cell damage by oxidative stress [14, 17]. It is noteworthy that these beneficial effects on improvement of cellular damage were improved by metformin in a time dependent manner. As was also found by other authors [10, 28], the current study demonstrated the increased MDA levels and the reduction of SOD activity in the EG treated group compared with controls, while these parameters reversed significantly in the renal tissue of rats cotreated with oxalate and metformin. Although ROS are mainly produced through the involvement of both mitochondria and NADPH oxidase, NADPH oxidase is a major source of ROS in the kidneys [27]. Although still experimental, metformin has been found to be particularly useful in the inhibition of NADPH oxidase, with strikingly high sensitivity and specificity [14]. Therefore, the fact that metformin diminished lipid peroxidation suggests that protection conferred by biguanide occurs from within renal cells and rat kidney tissue. It is noteworthy that SOD activity in the EG + metformin treated group was slightly higher than in the EG treated group but this was not statistically significant. However, when considering the MDA level, this study can still clearly indicate that metformin attenuates free radical injury induced by oxalate or hyperoxaluria in vitro and in vivo.

There was no difference in serum biochemistry between the experimental groups except that a significant slight increase of serum phosphorus in the EG treated group was observed in comparison to the control group. These results are consistent with previous serum biochemistry study in the EG-induced rat model [29]. As regards the urine examination of stone model experiment, metformin decreased the urinary calcium and oxalate in spite of a significant increase in the metformin group versus controls. These findings in the present study were similar to that seen in previous studies [28, 29]. From these results, we speculate that changes in the urinary biochemistry are one of the causes of decreased crystal formation in the kidney of rats cotreated with oxalate and metformin.

Another potentially important finding of this study was that crystal formation was considerably prevented in the metformin group compared to the stone forming group. Consequently, these results well confirmed previous work that antioxidants can ameliorate urolithiasis, particularly for NADPH oxidase inhibitors [9–11, 28, 30]. As Khan stressed, the inhibitor of NADPH oxidase is an efficient target for renal injury caused by oxalate and calcium oxalate crystals due to hyperoxaluria [30, 31].

This study inevitably has certain potential limitations. (1) The first one is renal crystal deposition caused by hyperoxaluria in the EG-induced rat model. This is different from patients with CaOx stones but may occur in patients with primary hyperoxaluria. (2) The serum concentration of metformin was not assessed in the present study. In vitro studies have shown that high concentrations of metformin could induce cell viability inhibition. (3) A dose of 200 mg/kg/day metformin was administered in in vivo experiment as recommended by previous study [23]. However, we do not know the relationship between metformin levels in rats and humans. Therefore, we suggest that further clinical trials should be performed so as to identify the proper level of metformin in serum which may prevent renal stone formation in humans.

## 5. Conclusions

Overall, it can be concluded from our present investigation that metformin has potent antioxidant effects and could effectively reduce renal tubular injury resulting from lipid peroxidation production induced by oxalate. Additionally, to the best of our knowledge, this report is the first to show that metformin inhibits renal crystal deposition in rats. These findings establish metformin as a new prospective therapeutic agent for treatment of calcium oxalate stone formation and might benefit individuals with primary hyperoxaluria or recurrent calcium oxalate stone formation.

## Figures and Tables

**Table 1:** Serum and urinary biochemistry.

	Mean ± SD		
	Control	EG	EG + metformin
Serum (mg/dL)			
Creatinine	0.32 ± 0.03	0.35 ± 0.05	0.33 ± 0.05
Ca	10.34 ± 0.41	10.61 ± 0.46	10.29 ± 0.20
P	6.69 ± 0.46	7.40 ± 0.46^*∗*^	7.01 ± 0.34
Mg	2.62 ± 0.42	2.45 ± 0.50	2.55 ± 0.37
Urine			
Volume (mL)	22.07 ± 3.31	25.02 ± 5.18	24.47 ± 4.05
PH	7.57 ± 0.46	7.40 ± 0.26	7.30 ± 0.30
Ca (mg/day)	3.32 ± 0.39	2.98 ± 0.37	2.60 ± 0.55^*∗*^
Ox (mg/day)	1.68 ± 0.57	18.62 ± 2.42^*∗*^	14.95 ± 1.49^*∗*#^

^*∗*^ *P* < 0.05, the control group versus the EG treated group and the EG + metformin treated group.

^#^ *P* < 0.05, the EG treated group versus the EG + metformin treated group.
